# Molecular Epidemiology of Hantaviruses in the Czech Republic

**DOI:** 10.3201/eid2511.190449

**Published:** 2019-11

**Authors:** Hana Zelena, Petra Strakova, Marta Heroldova, Jakub Mrazek, Tomas Kastl, Alena Zakovska, Daniel Ruzek, Jan Smetana, Ivo Rudolf

**Affiliations:** Institute of Public Health in Ostrava, Ostrava, Czech Republic (H. Zelena, J. Mrazek);; University of Defence, Hradec Kralove, Czech Republic (H. Zelena, J. Smetana);; The Czech Academy of Sciences, Brno, Czech Republic (P. Strakova, M. Heroldova, I. Rudolf);; Veterinary Research Institute, Brno (P. Strakova, T. Kastl, D. Ruzek); Mendel University, Brno (M. Heroldova);; Masaryk University, Brno (A. Zakovska);; Biology Centre of the Czech Academy of Sciences, Ceske Budejovice, Czech Republic (D. Ruzek)

**Keywords:** Dobrava, Kurkino, hantavirus, rodents, patients, RT-PCR, Czech Republic, viruses, molecular epidemiology, Puumala virus, orthohantavirus, Dobrava-Belgrade virus, Tula virus, Seewis virus

## Abstract

During 2008–2018, we collected samples from rodents and patients throughout the Czech Republic and characterized hantavirus isolates. We detected Dobrava-Belgrade and Puumala orthohantaviruses in patients and Dobrava-Belgrade, Tula, and Seewis orthohantaviruses in rodents. Increased knowledge of eco-epidemiology of hantaviruses will improve awareness among physicians and better outcomes of patients.

The most prevalent hantaviruses in Europe are Tula virus, Puumala virus (PUUV), and Dobrava-Belgrade virus (DOBV), all orthohantaviruses; PUUV and DOBV cause hemorrhagic fever with renal syndrome (*1*). Four DOBV genotypes of different virulences in humans are known: the nonpathogenic Saaremaa; Kurkino, which causes mostly mild disease; and Dobrava and Sochi, which are both highly pathogenic (*2*).

Tula virus is the most frequently detected hantavirus in rodents in the Czech Republic, followed by PUUV in Moravia and DOBV in South Bohemia. The seroprevalence of hantaviruses in humans in the Czech Republic is 1%–1.4% (*3*). In 2009, one case of DOBV infection in a hospitalized patient was reported in the Czech Republic (*4*), and in 2011, two more occurred at the Czech Republic–Slovakia border (*5*); in 2017, a fatal DOBV case was reported (*6*). All 4 of these cases were classified as Dobrava genotype by PCR and sequencing. Overall, 82 hantavirus infections were reported in humans in the Czech Republic during 2008–2017 (*7*). In this study, we aimed to determine the location of DOBV reservoirs in the Czech Republic and molecularly characterize positive samples.

During 2010–2017, we collected 1,551 wild rodents from different locations of the Czech Republic: 618 yellow-necked mice (*Apodemus flavicollis*), 37 wood mice (*A. sylvaticus*), 222 striped field mice (*A. agrarius*), 445 bank voles (*Myodes glareolus*), 40 common voles (*Microtus arvalis*), and 189 field voles (*Microtus agrestis*). We trapped all rodents as specified by the Animal Protection Act No. 246/1992 of the Czech Republic. Moreover, we obtained 61 clinical samples acquired from patients with hantavirus infections during 2008–2018; these hantavirus diagnoses were based on serologic testing (detection of hantavirus-specific IgG and IgM) and clinical and laboratory findings (fever, renal dysfunction, thrombocytopenia).

We tested human serum samples by Anti-Hantavirus Pool 1 “Eurasia” ELISA (IgG and IgM) and confirmed previous hantavirus results by immunoblot EUROLINE Anti-Hanta Profile 1 (IgG and IgM) (both Euroimmun, https://www.euroimmun.com). We isolated RNA from rodent lung samples and human serum, plasma, and whole blood samples using QIAamp Viral RNA Kit (QIAGEN, https://www.qiagen.com) and QIAzol (QIAGEN) or TRIzol (Invitrogen, https://www.thermofisher.com). We screened rodent and human RNA samples for hantavirus RNA using a reverse transcription PCR that amplified a 390-bp fragment of the large (L) segment (*8*). We tested L segment–positive samples with additional PCRs targeting regions of the medium (M) and small (S) segments (*4*). We analyzed hantavirus sequences using BLAST (https://blast.ncbi.nlm.nih.gov/Blast.cgi), aligned with BioEdit (*9*), and built phylogenetic trees using MEGA 7.0 (https://megasoftware.net).

Of 1,551 rodent samples, 43 (2.77%) were PCR positive for hantavirus. These 43 animals were from 3 sampled localities: Celadna in the Beskydy Mountain region (in 2010, n = 9), Petrovice u Karviné (in 2014, n = 33), and Velká Stolová Mountain (in 2014, n = 1). From 9 hantavirus-positive mice collected in 2010, we recovered 6 sequences: 2 identical sequences of DOBV Dobrava in 2 *A. flavicollis* mice (GenBank accession no. MK605679) and 4 sequences (2 identical) of Seewis virus in 3 *A. flavicollis* mice and 1 *A. sylvaticus* mouse (GenBank accession nos. MK605682–4). The Seewis virus sequences clustered with sequences derived from shrews from the Beskydy Mountain region (GenBank accession nos. JQ425316, JQ425337, JQ425340; data not shown). From 33 positive *A. agrarius* mice trapped in Petrovice u Karviné, we recovered 10 Kurkino genotype sequences with 99.1%–100% similarity (GenBank accession nos. MK605680–1). We detected Tula virus (GenBank accession no. MK605685) in 1 field vole trapped at Velká Stolová Mountain.

Of 61 seropositive patients, 32 were PCR positive for DOBV and 3 PCR positive for PUUV (acquired outside of the Czech Republic). We recovered partial L segment sequences from 28 of 32 DOBV-positive patients (GenBank accession nos. MK605641–65). Because of low quality, we manually shortened these sequences to 195 bp. We obtained partial (264-bp) M segment sequences from 6 patients (GenBank accession nos. MK605666–71) and partial (531-bp) S segment sequences from 7 patients (GenBank accession nos. MK605672–8). We constructed phylogenetic trees to compare the virus sequences from humans and rodents. Analysis of the L segment revealed that samples clustered into 2 separate groups by DOBV genotype (Dobrava or Kurkino), and virus sequences from the same areas (regardless of human or rodent origin) clustered into the same clades (Figure). Clustering by DOBV genotype was also observed in phylogenetic trees constructed with the M and S segments (data not shown).

**Figure:**
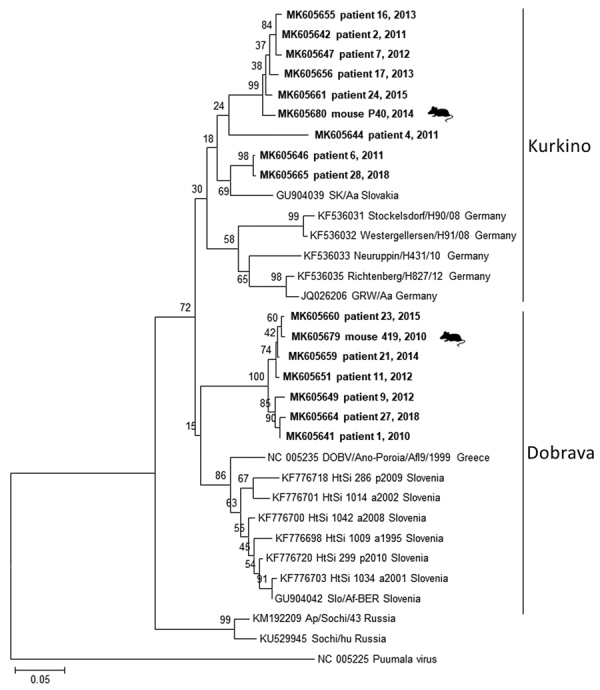
Phylogenetic tree constructed with partial 195-bp fragments of the DOBV large segment from humans and mice, Czech Republic, 2010–2018. Sequences from this study (bold) were compared with available sequences from the GenBank database; patient numbers are provided, and mouse samples are labeled. Samples with sequences identical to another sample were excluded for simplification purposes. Sequences were aligned with BioEdit (*9*), and the phylogenetic tree was prepared by using MEGA 7.0 (https://megasoftware.net) and the neighbor-joining method. Analyses were performed with the Jukes-Cantor model by using a gamma distribution with 5 rate categories and a bootstrap value of 1,000. The genotype clusters are labeled. Scale bar indicates nucleotide substitutions per site. The Puumala virus sequence was used as an outgroup. DOBV, Dobrava-Belgrade virus.

Most Dobrava-positive patients were from the mountain regions Jeseniky (northwest Silesia) and Beskydy (south Silesia), whereas Kurkino cases occurred in the lowlands between these 2 mountain regions (Appendix Figure). The only confirmed fatal DOBV case in the Czech Republic was in a patient living in Kladno District, Central Bohemia region (*6*). The geographic distribution of DOBV genotypes seems to be linked with the distribution of *Apodemus* spp. mice (*10*). The higher number of hantavirus cases in Silesia might be caused by an increased prevalence of DOBV in rodents or could be the result of an increased awareness of DOBV among local physicians.

Appendix Additional information on molecular epidemiology of hantaviruses in the Czech Republic.

## References

[1] Olsson GE, Leirs H, Henttonen H. Hantaviruses and their hosts in Europe: reservoirs here and there, but not everywhere? Vector Borne Zoonotic Dis. 2010;10:549–61. 10.1089/vbz.2009.0138 20795916

[2] Klempa B, Avsic-Zupanc T, Clement J, Dzagurova TK, Henttonen H, Heyman P, et al. Complex evolution and epidemiology of Dobrava-Belgrade hantavirus: definition of genotypes and their characteristics. Arch Virol. 2013;158:521–9. 10.1007/s00705-012-1514-5 23090188 PMC3586401

[3] Pejcoch M, Kríz B. Hantaviruses in the Czech Republic. Emerg Infect Dis. 2003;9:756–7. 10.3201/eid0906.020772 12781027 PMC3000151

[4] Papa A, Zelená H, Barnetová D, Petrousová L. Genetic detection of Dobrava/Belgrade virus in a Czech patient with Haemorrhagic fever with renal syndrome. Clin Microbiol Infect. 2010;16:1187–90. 10.1111/j.1469-0691.2009.03075.x 19832712

[5] Zelena H, Zvolankova V, Zuchnicka J, Liszkova K, Papa A. Hantavirus infection during a stay in a mountain hut in Northern Slovakia. J Med Virol. 2011;83:496–500. 10.1002/jmv.21984 21264871

[6] Zelená H, Rumlerová M, Kodras K, Beroušková P, Mrázek J, Smetana J. [Hantavirus causing fatal haemorrhagic fever in the Czech Republic] [in Czech]. Epidemiol Mikrobiol Imunol. 2017;66:149–52. 28948811

[7] Státní Zdravotní Ústav. Cases of selected infectious diseases in the Czech Republic, January–July 2019. 2019 [cited 2019 Sep 10]. http://szu.cz/publikace/data/2019/vyskyt-vybranych-hlasenych-infekci-v-ceske-republice-leden-1?lang=1

[8] Klempa B, Fichet-Calvet E, Lecompte E, Auste B, Aniskin V, Meisel H, et al. Hantavirus in African wood mouse, Guinea. Emerg Infect Dis. 2006;12:838–40. 10.3201/eid1205.051487 16704849 PMC3374458

[9] Hall TA. BioEdit: a user-friendly biological sequence alignment editor and analysis program for Windows 95/98/NT. Nucleic Acids Symp Ser. 1999;41:95–8.

[10] Sibold C, Ulrich R, Labuda M, Lundkvist A, Martens H, Schütt M, et al. Dobrava hantavirus causes hemorrhagic fever with renal syndrome in central Europe and is carried by two different *Apodemus* mice species. J Med Virol. 2001;63:158–67. 10.1002/1096-9071(20000201)63:2<158::AID-JMV1011>3.0.CO;2-# 11170053

