# Supplementary material for: Molecular Epidemiology of Hantaviruses in the Czech Republic
Source: Emerg Infect Dis. 2019 Nov;25(11):2133–5. doi: 10.3201/eid2511.190449 (PMC6810203; doi:10.3201/eid2511.190449)
Supplement: Appendix — Additional information on molecular epidemiology of hantaviruses in the Czech Republic. [file 19-0449-Techapp-s1.pdf]

Article DOI: https://doi.org/10.3201/eid2511.190449

Molecular Epidemiology of Hantaviruses in the Czech Republic

Appendix

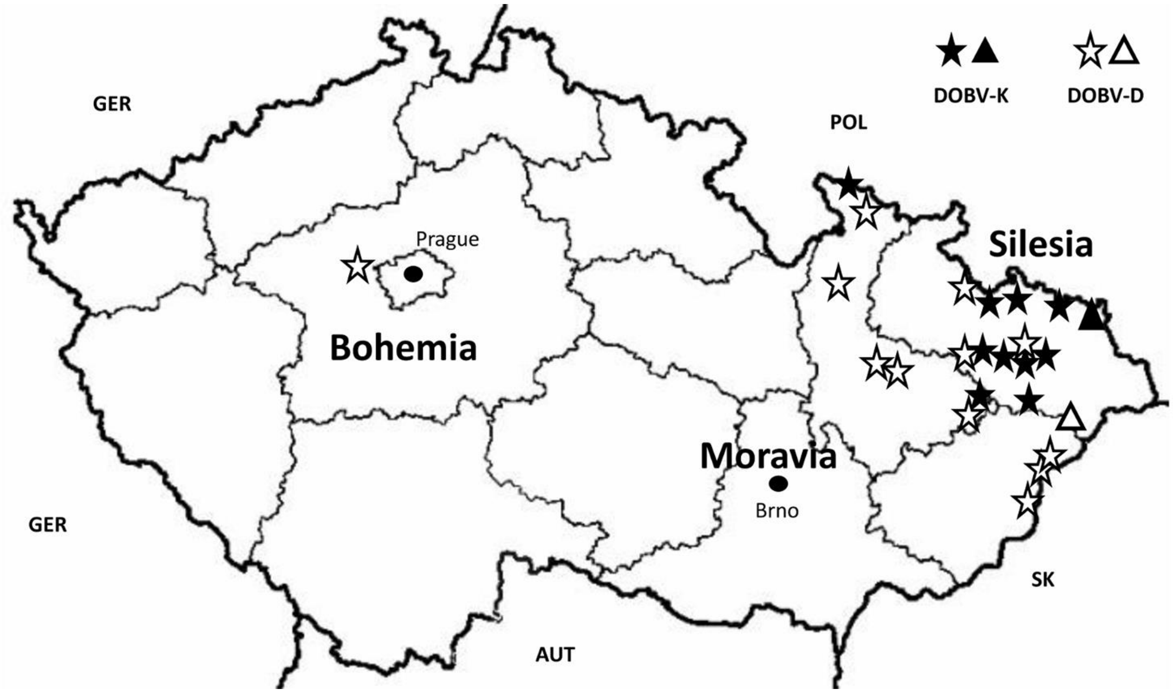

Appendix Figure. Map of the Czech Republic with given localities of DOBV-positive patients and wild rodents to show the distribution of genotypes Dobrava and Kurkino. Black triangle indicates Kurkino genotype in mice. White triangle indicates Dobrava genotype in mice. Black star indicates Kurkino genotype in patients. White star indicates Dobrava genotype in patients. Locations and coordinates of hantavirus-positive patients and the corresponding virus genotypes: Jesenik (50°13′47″N 17°12′17″E; 1 DOBV-D, 1 DOBV-K); Sumperk (49°57′55″N 16°58′15″E; 1 DOBV-D); Olomouc (49°35′38″N 17°15′03″E; 2 DOBV-D); Prerov (49°27′20″N 17°27′4″E; 1 DOBV-D, 2 DOBV-K); Bruntal (49°59′N 17°27′E; 1 DOBV-D); Opava (49°56′N 17°54′E; 2 DOBV-K); Ostrava (49°50′08″N 18°17′33″E; 3 DOBV-K, 1 DOBV-D); Novy Jicin (49°35′N 18°00′E; 1 DOBV-D, 4 DOBV-K), Karvina (49°51′15″N 18°32′34″E; 1 DOBV-K), Vsetin (49°21′N 18°00′E; 1 DOBV-D), Zlin (49°14′N 17°40′E; 1 DOBV-D), and Kladno (50°9′N 14°6′E 1 DOBV-D). Locations and coordinates of hantavirus-positive rodents with the corresponding virus genotypes: Celadna, district Frydek Mistek (49°32′51″N 18°20′14″E; 3 DOBV-D in *Apodemus flavicollis*, 1

Seewis hantavirus in *A. sylvaticus*), Petrovice u Karvine, district Karvina (49°53′05.2”N 18°31′49.9”E; 33 DOBV-K in *A. agrarius*), and Velka Stolova, district Frydek Mistek (49°32′55.4”N 18°20′15.3”E; 1 Tula orthohantavirus in *Microtus agrestis*). Seewis orthohantavirus and Tula orthohantavirus are not shown in the phylogenetic tree (Figure, main text) or on the map. AUT, Austria; DOBV-D, Dobrava-Belgrade orthohantavirus Dobrava; DOBV-K, Dobrava-Belgrade orthohantavirus Kurkino; GER, Germany; POL, Poland; SK, Slovakia.
